# Energy Expenditure Improved Risk Factors Associated with Renal Function Loss in NAFLD and MetS Patients

**DOI:** 10.3390/nu13020629

**Published:** 2021-02-15

**Authors:** Manuela Abbate, Catalina M. Mascaró, Sofía Montemayor, María Barbería-Latasa, Miguel Casares, Cristina Gómez, Escarlata Angullo-Martinez, Silvia Tejada, Itziar Abete, Maria Angeles Zulet, Antoni Sureda, J. Alfredo Martínez, Josep A. Tur

**Affiliations:** 1 Research Group in Community Nutrition and Oxidative Stress, University Research Institute of Health Sciences (IUNICS), University of the Balearic Islands, 07122 Palma de Mallorca, Spain; manuela.abbate@uib.es (M.A.); c.mascaro@uib.es (C.M.M.); sofiamf16@gmail.com (S.M.); eangullo@yahoo.es (E.A.-M.); silvia.tejada@uib.es (S.T.); antoni.sureda@uib.es (A.S.); 2 Health Research Institute of Balearic Islands (IdISBa), 07120 Palma de Mallorca, Spain; 3 Department of Preventive Medicine and Public Health, University of Navarra, 31008 Pamplona, Spain; mbarberia.3@alumni.unav.es; 4 Radiodiagnosis Service, Red Asistencial Juaneda, 07011 Palma de Mallorca, Spain; casaresmiguel@gmail.com; 5 Clinical Analysis Service, University Hospital Son Espases, 07120 Palma de Mallorca, Spain; cristina.gomez@ssib.es; 6 Escola Graduada Primary Health Care Center, 07002 Palma de Mallorca, Spain; 7 CIBER Physiopathology of Obesity and Nutrition (CIBEROBN), Instituto de Salud Carlos III (ISCIII), 28029 Madrid, Spain; iabetego@unav.es (I.A.); mazulet@unav.es (M.A.Z.); jalfredo.martinez@imdea.org (J.A.M.); 8 Department of Nutrition, Food Sciences, and Physiology, Center for Nutrition Research, University of Navarra, 31008 Pamplona, Spain; 9 Cardiometabolics Precision Nutrition Program, IMDEA Food, CEI UAM-CSIC, 28049 Madrid, Spain

**Keywords:** glomerular hyperfiltration, albumin-to-creatinine ratio, non-alcoholic fatty liver disease, caloric restriction, increased energy expenditure

## Abstract

To assess the efficacy of three lifestyle interventions on the reduction of liver fat content and metabolic syndrome (MetS), and whether such reductions would influence renal outcomes, we conducted a randomized controlled trial on 128 participants with MetS and non-alcoholic fatty liver disease (NAFLD), as well as available data on estimated glomerular filtration rate (eGFR) and urinary albumin-to-creatine ratio (UACR). Patients were randomized in 1:1:1 ratio to either Conventional Diet, Mediterranean diet (MD)–high meal frequency, and MD–physical activity groups. Each intervention aimed at reducing caloric intake by 25%–30% of baseline intake and increase energy expenditure by 400 kcal/70 kg. Patients attended regular visits and were followed-up for 6 months. Increased albuminuria was present in 13.3% of patients, while 32.8% showed hyperfiltration. UACR reduction was associated with higher levels of UACR at baseline but not with changes in liver fat. eGFR decreased in patients presenting hyperfiltration at baseline and was associated with reduction in liver fat and insulin resistance, as well as with increase in energy expenditure (R^2^ = 0.248, *p* = 0.006). No significant differences were observed between the three treatment groups. In patients with NAFLD and MetS, energy expenditure significantly reduced hepatic fat accumulation and insulin resistance, which reduced glomerular hyperfiltration. Increased albuminuria was reduced, but it was not associated with reduced liver fat.

## 1. Introduction

Non-alcoholic fatty liver disease (NAFLD), defined as the presence of excessive hepatic fat accumulation in patients with no previous history of alcohol abuse [1], has been associated with an increased risk of cardiovascular disease, cardiovascular mortality, and all-cause mortality, especially in patients presenting concomitant metabolic abnormalities [2]. NAFLD also seems to increase the risk of chronic kidney disease (CKD) [3], and although NAFLD and CKD share multiple cardiometabolic abnormalities [4], consistent evidence showed that NAFLD may precede CKD and be an important risk factor for its development [5]. 

CKD is defined by a reduced glomerular filtration rate (GFR) (<60 mL/min/1.73 m^2^) and/or kidney damage (indicated by albuminuria >30 mg/g or proteinuria) [6]. Importantly, especially in metabolically compromised obese patients, CKD is often preceded by a phase of glomerular hyperfiltration [4,7], a risk factor for accelerated renal function loss and albumin ultrafiltration and excretion [8,9]. Amelioration of hyperfiltration using renin-angiotensin-aldosterone system (RAAS) inhibitors [8] or weight loss [9,10] has been observed to offer reno-protective effects in patients with type 2 diabetes (T2DM) by significantly slowing down long-term GFR decline similar to that of healthy aging adults. 

So far, the association between NAFLD and glomerular hyperfiltration has only been explored in obese children [11]. The article concluded that hyperfiltration was associated with greater NAFLD activity score, independent of age, sex, ethnicity, obesity severity, T2DM, and medications. In adults, such association has not been explored yet; however, since NAFLD is a driver of CKD [5], and hyperfiltration is often defined as the first stage of renal impairment [12], it could be speculated that amelioration of NAFLD could prevent CKD in its most primordial stage. 

Weight loss through calorie restriction and increased energy expenditure is the only currently available strategy for treating NAFLD in patients with metabolic syndrome (MetS) [1]. Weight loss directly reduces hepatic fat accumulation, while concomitantly ameliorating other cardiometabolic risk factors associated with NAFLD and its progression to more advanced stages. Consequently, reduced hepatic fat accumulation and an improved metabolic state achieved through weight loss intervention may influence changes in GFR and urinary albumin excretion in patients with MetS. 

The aim of this study was to assess the efficacy of lifestyle intervention on the reduction of liver fat content and MetS, along with whether such reductions would influence renal outcomes.

## 2. Materials and Methods

### 2.1. Study Design

The current randomized controlled trail proposed a personalized nutritional intervention based on a Mediterranean customized diet [13], coupled with physical activity promotion, to prevent and reverse NAFLD among obese patients with metabolic syndrome. The study is registered at ClinicalTrials.gov with number NCT04442620 (https://clinicaltrials.gov/ct2/show/NCT04442620; accessed on 14 February 2021). The study protocol was reviewed and approved by the Ethics Committee of the Balearic Islands (ref. IB 2251/14 PI; approved on 26 February 2020) and the Ethics Committee of the University of Navarra (ref. 054/2015mod2; approved on 22 February 2018), and it followed the Declaration of Helsinki ethical standards. All participants were informed of the purpose and the implications of the study and provided the written consent to participate.

### 2.2. Subjects

Participants were male and female individuals who satisfied all eligibility criteria. Inclusion criteria included: aged 40 to 60 years, previous diagnosis of NAFLD by liver ultrasound, Body Mass Index (BMI) between 27 and 40 kg/m^2^, and presenting at least three of the five MetS traits as described in the International Diabetes Federation (IDF) consensus [14]: (1) BMI >30 kg/m^2^ or increased waist circumference: ≥94 cm in males and ≥80 cm in females; (2) triglycerides (TG) levels ≥ 150 mg/dL (1.7 mmol/L), or specific treatment; (3) reduced high-density lipoprotein cholesterol (HDL-C): <40 mg/dL (1.03 mmol/L) in males and <50 mg/dL (1.29 mmol/L) in females, or specific treatment; (4) raised blood pressure (BP): systolic BP ≥130 or diastolic BP ≥ 85 mm Hg, or treatment of previously diagnosed hypertension; (5) raised fasting plasma glucose (FPG) ≥100 mg/dL (5.6 mmol/L), or previously diagnosed type 2 diabetes (T2DM). Exclusion criteria: previous cardiovascular disease, liver disease (other than NAFLD), cancer or a history of malignancy in the previous 5 years, haemochromatosis, previous bariatric surgery, non-medicated depression, alcohol and drug abuse, pregnancy, primary endocrinological diseases (other than non-medicated hypothyroidism), concomitant therapy with steroids, or inability to provide informed consent.

From June 2018 to January 2020, 237 patients were screened for eligibility. Of those, 82 did not meet inclusion criteria or refused to participate. Finally, 155 patients were randomized in a 1:1:1 ratio to one of the three intervention groups. Randomization was carried out using the MinimPy desktop minimization program, and patients were stratified by gender (male/female), T2DM (yes/no), and stage of steatosis (mild/moderate/severe). The randomization process was performed by a dedicated person and blinded to all staff and the principal investigator. 

Participants were randomly allocated to one of the following three groups: 

(1) The Conventional Diet (CD) group, which followed the American Association for the Study of Liver Disease (AASLD) recommendations [1] with energy restriction enough to lose 3%–5% of body weight to improve steatosis, and 7%–10% to improve most of the histopathological features of Non-Alcoholic SteatoHepatitis (NASH), including fibrosis, following the general guidelines of the U.S. Department of Health and Human Services and U.S. Department of Agriculture (20%–35% fat, 10%–35% protein, 45%–65% carbohydrate) [15]. 

(2) The Mediterranean Diet–high meal frequency (MD-HMF) group, which was instructed to adhere to a Mediterranean Diet based on a distribution of macronutrients of 30%–35% fat (mainly mono- and poly-unsaturated fatty acids from extra virgin olive oil, nuts, and omega-3 containing foods), 25% protein (mainly from vegetable sources), and 40%–45% carbohydrates (50%–70% of the total carbohydrate intake should low on glycaemic index and rich in fiber). This diet was previously observed to reduce fat mass and overall weight and improve general oxidative stress in patients with the metabolic syndrome [16], providing high Total Antioxidant Capacity (TAC), and focused on the chronological distribution of meals, as factors, such as meal frequency and distribution could aid in reducing the feeling of hunger, thus improving compliance to an energy restricted dietary regime [17]. The total daily caloric intake of this diet was distributed over seven meals, with the highest calorie meals to be consumed early during the day.

(3) The Mediterranean Diet–physical activity (MD-PA) group, which followed an energy-restricted Mediterranean diet. Meal frequency would be of 4–5 meals a day including snacks. This group consumed 35%–40% of total calories from fat (8–10% of Saturated Fatty Acids, >20% of Monounsaturated Fatty Acids, >10% of Polyunsaturated Fatty Acids and <300 mg/day of Cholesterol), approximately 20% of total calories from proteins and 40–45% or more of total calories from carbohydrates (low glycaemic index). Sodium chloride should not exceed 6 g a day (2.4 g of sodium), and dietary fiber should be no less than 30–35 g/day [18].

Trained dietitians provided patients in the three arms with a prescription of total calories to consume daily, dietary plans based on exchange systems, and a 7-day menu for each season. Patients were advised to monitor their weight weekly, and, to facilitate adherence, patient-dietitian contact (in person, by telephone, or e-mail) was provided once every two weeks during the first 6 months and once a month thereafter.

In terms of physical activity, the CD and MD-HMF groups were instructed to accumulate a minimum of 10,000 steps a day (recorded by a personal pedometer) [19], while the MD-PA group was instructed to undergo 35 min interval training session three times a week, in the combination of two instructor-led on-site training and one remote prescribed training session a week for the whole duration of the trial. The 35-min on-site training sessions were divided in three different phases: a 5-min warm-up, 20-min interval training, and 10-min breathing and stretching. The interval training included five activities of moderate intensity with adequate recovery between sets. The “remote” training sessions, on the other hand, were pre-recorded video sessions that patients would receive via instant messages or e-mails. The weekly moderate-intensity aerobic physical activity proposed was equivalent to 10,000 daily steps in terms of caloric expenditure (400 kcal for a person that weights 70 kg); nevertheless, the intensity of each exercise was adjusted to the physical condition of each subject. Fitness specialists were responsible for the content of both the on-site and remote training sessions and provided training and support to volunteering research team members giving interval training sessions to patients.

Each intervention aimed at reducing caloric intake by 25%–30% of baseline intake and increase energy expenditure by 400 kcal/70 kg (5.7 kcal per kg of body weight). 

### 2.3. Measurements

Information on sociodemographic, medical history, smoking status, and alcohol consumption was collected by a dietitian and study nurse at baseline. Height was also measured at baseline using a mobile stadiometer (Seca 213, SECA Deutschland, Hamburg, Germany), with the participant’s head maintained in the Frankfort plane, and to the nearest millimeter.

At baseline and at 6-month follow-up, weight, body fat, BMI, waist circumference (WC), blood pressure (BP), and information on energy expenditure were taken by trained dietitians. Weight and body fat were measured using a Segmental Body Composition Analyzer for impedance testing (Tanita MC780P-MA, Tanita, Tokyo, Japan), with participants wearing light clothes and no shoes (0.6 kg of weight was subtracted for their clothing). BMI was calculated using the standard formula (weight in kilograms divided by the square of height in meters). WC was measured in duplicate with an anthropometric tape, halfway between the last rib and the iliac crest, with participants standing upright. BP was measured in triplicate (2 min apart) with a validated semi-automatic oscillometer (Omron HEM-705CP, Hoofddorp, The Netherlands), in the non-dominant arm after a 5-min rest in a seated position. The average of the three measurements was recorded and used for statistical analysis. Information on mean weekly time (in minutes) of physical activity was collected using the Minnesota Leisure Time Physical Activity Questionnaire (Spanish version): energy expenditure was expressed as metabolic equivalents of task (MET)·min/week [20,21].

### 2.4. Dietary Intakes

Mean dietary intakes at baseline and 6-months were assessed by a trained dietitian using a validated 148 items-Food Frequency Questionnaire (FFQ) [22]. The 148 items consist of usual portion sizes of foods and beverages with response categories to indicate frequency of consumption over a period of 12 months. Energy and nutrients intakes were calculated by multiplying the nutrient composition of the portion size of each item by the frequency of consumption using a computer program based on available food composition tables [23]. Dietary information derived from the 148-items FFQ included total energy expressed as kcal per day (kcal/d), macro- and micro-nutrient intakes, and intakes according to food groups.

Adherence to the MedDiet was assessed by means of a 17-items MedDiet adherence questionnaire, previously used in the PREvención con DIeta MEDiterránea/Prevention with Mediterranean Diet (PREDIMED) trial [24]. Each item of the questionnaire related to a specific dietary objective contemplated by the MedDiet and could be scored as 1 (compliance) or 0 (non-compliance). The total score ranged between 0 and 17, such as a score of 0 indicated no compliance, and a score of 17 indicated maximum adherence.

### 2.5. Blood Collection and Analysis

At each visit, venous blood and single spot urine samples were collected in the morning after a 12-h overnight fast. Blood was collected through a venous catheter from the antecubital vein in suitable vacutainers. Measures included routine laboratory parameters, such as fasting glucose, glycated hemoglobin (HbA1c), bilirubin, aspartate aminotransferase (AST), alanine aminotransferase (ALT), gamma-glutamyl transferase (GGT), uric acid, urea, creatinine, albumin, total cholesterol, high-density lipoprotein cholesterol (HDL-C), and triglyceride (TG). Low-density lipoprotein cholesterol (LDL-C) was calculated according to the Friedewald Formula [25]. Additional measures included serum fasting insulin, C-reactive protein (CRP), serum ferritin, and thyroid stimulating hormone (TSH). Urinary albumin excretion was measured as urinary albumin-to-creatine ratio (UACR). The urinary albumin concentration was determined by immunoturbidimetric assay and urinary creatinine concentration was measured by a modified Jaffe method on an Abbott ARCHITECT c16000 (Abbott, Abbott Park, Illinois, USA). Insulin resistance index was calculated using the Homeostatic Model Assessment for Insulin Resistance (HOMA-IR) formula by Matthews et al. [26], as well as the TGs and glucose (TyG) index, calculated as the natural logarithm of the product of fasting plasma glucose and TG [27].

Estimated GFR (eGFR) was calculated using the Chronic Kidney Disease Epidemiology Collaboration (CKD-EPI) formula developed in 2009 [28]. The equation normalizes estimated renal function for body surface area (BSA) and expresses as eGFR mL/min/1.73 m^2^. Although the equation has been validated in populations with normal, as well as low, GFR and is generally well-accepted [29], it has been argued that indexing eGFR for BSA in patients with increased weight can result in an underestimation of GFR, as well as masking a genuine association between renal function and body fat, such that it has been suggested that absolute estimates of GFR should be used instead [30,31,32]. Accordingly, eGFR was converted to absolute values (mL/min) by using the following formula [30]: (eGFR mL/min/1.73 m^2^ *BSA)/1.73 m^2^. BSA was calculated using the DuBois and DuBois equation [33]. Glomerular hyperfiltration was defined as eGFR ≥ 120 mL/min [34].

### 2.6. Magnetic Resonance Imaging (MRI)

Randomized patients presented a diagnosis of NAFLD by ultrasound at baseline, nevertheless presence of liver fat was verified by abdominal MRI (Signa Explorer 1.5T, General Electric Healthcare, Chicago, IL, USA, or Siemens Aera 1.5T, Siemens Medical Systems, Erlangen, Germany; depending on the recruiting center) and quantified as mean percentage (%). A mean intrahepatic fat ≥6.4% was considered clinically relevant [35].

### 2.7. Study Outcomes

The primary outcome was to assess changes in UACR and eGFR after a 6-months intervention for the whole sample and differences in change between the three intervention arms and within each arm. Changes in mean hepatic fat accumulation (quantified by MRI), as well as in anthropometric, clinical, laboratory, and dietary parameters, were also assessed. Lastly, possible relationships between these changes and changes in UACR and eGFR were also explored.

### 2.8. Statistics

Sample size was estimated for weight loss as the primary outcome of the study, assuming a two-group t-test (two-sided) of the difference between the control group and the two intervention groups (group ratio = 2). Based on previous evidence [16,36], a weight reduction difference of 2.5 kg with a standard deviation (SD) of 4.5 was expected between the control group and the intervention groups. A total sample size of 150 patients was needed to give the trial a 95% power to detect a statistically significant difference in weight loss between the control and the intervention groups (α = 0.05), as well as to account for a 20% drop-out rate. The analysis was conducted by modified intention to treat (mITT) with randomized participants analyzed according to the treatment group originally assigned but including only those with available UACR and eGFR data at both baseline and 6-months follow-up. Variable distribution was assessed by Shapiro-Wilk test and visual inspection of histograms and normal probability plots. Highly skewed variables, such as baseline and 6-months UACR, HOMA-IR, serum ferritin, fasting glucose, and triglycerides, were log-transformed before analysis; however, in the tables, they are presented as untransformed data for ease of interpretation. Continuous variables were expressed as means ± SD. Categorical data were expressed as count (%). One-way analysis of variance (ANOVA), or unequal variance t-test in case of heterogeneity for continuous variables were used to compare unadjusted means of baseline clinical characteristics between the three intervention groups to assess whether, by removing patients without available UACR and/or eGFR, the balance in baseline characteristics had been affected. Within-group comparisons before and after intervention, for the whole sample, were assessed by paired sample t-test. Within-group comparisons for each intervention were assessed by paired sample t-test and repeated measures ANOVA for continuous variables and by McNemar test for frequencies. Between-group changes were assessed by one-way ANCOVA, while statistically controlling for baseline measures. Post-hoc analyses were performed by applying the Bonferroni method. 

Bivariate correlations of changes (delta) in UACR and eGFR with changes (delta) in anthropometric, clinical, laboratory, and dietary parameters were evaluated by Pearson’s correlation coefficient. Variables with a level of significance (*p*) below 0.05 (two-tailed) were entered in multiple linear regression models to investigate the association between changes in independent covariates and changes in UACR/eGFR as outcome variables. The model was then adjusted for the delta of energy intake (kcal), BMI (kg/m^2^), systolic BP (mmHg), TyG, METs (min/week), and intervention group. All *p* values were two-sided, with *p* < 0.05. 

Statistical analysis was performed using SPSS statistical software package, version 25.0 (SPSS Inc., Chicago, IL, USA).

## 3. Results

Of the 155 patients included, 4 patients withdrew their consent before receiving intervention, and 8 withdrew their consent or were lost-to-follow-up before completing the first 6 months of the trial. Moreover, 14 patients did not present available data on UACR/eGFR at baseline and/or 6-months and were excluded from the analysis. One patient who showed evidence of proteinuria and reduced renal function (UACR = 3871.6 mg/g; eGFR = 23.3 mL/min/1.73 m^2^) was also excluded from the analysis to avoid biased results. Finally, data from a total of 128 participants were analyzed. Patients were distributed as follows: 42 in the CD group; 44 in the DM-HMF group; and 43 in the DM-PA group.

### 3.1. Patients’ Characteristics

As shown in Table 1, of the 128 patients, 50 (39.1%) were females, 24 (18.7%) were current smokers, 25 (19.5%) were consuming more than 7 alcoholic beverages a week, and most patients (53, 41.4%) did not practice physical activity. Mean age ± SD was 52.9 ± 7.4 years. At baseline, 29 participants (22.7%) were diabetics, and 53 (41.4%) showed hypertension. 

### 3.2. Effect of Intervention on Anthropometrics, Energy Expenditure, Adherence to Mediterranean Diet, UACR, and eGFR

Table 2 shows changes in anthropometrical, lifestyle, renal, and hepatic variables within and between intervention groups at 6 months versus baseline. At baseline, weight averaged 95.5 ± 14.7 kg, BMI averaged 33.6 ± 3.6 kg/m^2^, waist circumference was 111.8 ± 9.0 cm, and fat mass % was 35.7 ± 7.0. After intervention, body weight decreased by 6.1 ± 5.6 kg in the whole sample (*p* < 0.001), 5.7 ± 5.5 kg in the CD group (*p* < 0.001), 7.8 ± 6.0 kg in the MD-HMF group (*p* < 0.001), and 4.8 ± 5.0 kg in the MD-PA group (*p* < 0.001). These changes were different between the MD-HMF and MD-PA groups (*p* = 0.048). Time per group interaction for weight was also significant (*p* = 0.040). Consistent with the change in weight, BMI was also reduced for the whole sample, as well as for each group. Differences in BMI change between groups were not significant; nevertheless, the interaction between time and group was significant (*p* = 0.030). Waist circumference was reduced in the whole sample, as well as in the MD-HMF and the MD-PA groups, but not in the CD group. Fat mass %, on the other hand, did not change appreciably for the whole sample or for the MD-HMF group, but it reduced significantly in the CD and MD-PA groups. Time group interaction for waist circumference and fat mass % were not significant, as well as in waist circumference and percentage of fat mass change between groups.

At baseline, adherence to the MedDiet scored an average of 7 ± 3. After intervention, adherence to the MedDiet increased significantly for the whole sample, as well as for each intervention group. Between-group changes were significant between the CD and the MD-HMF groups and the MD-HMF and MD-PA groups (*p* < 0.001). Time per group interaction was also significant (*p* < 0.001). Physical activity increased significantly for the whole sample; however, they did not reach significance for any of the intervention groups. Changes between groups were also not significant. 

At baseline, UACR for the whole sample was 15.3 ± 31.8 mg/g, and 17 patients (13.3%) showed UACR values between 30 and 300 mg/g. eGFR was 102.9 ± 25.5 mL/min, and 42 participants (32.8%) showed hyperfiltration. Patients with UACR 30–300 mg/g were 6 (4.7%) in the CD group, 2 (1.6%) in the MD-HMF group, and 9 (7.0%) in the MD-PA group. Patients with eGFR > 120mL/min were 12 (9.7%) in the CD group, 9 (7.3%) in the MD-HMF group, and 15 (12.1%) in the MD-PA group. 

After 6-month intervention, UACR decreased significantly for the whole sample (−6.3 ± 26.5 mg/g; *p* < 0.001), for the CD group (−11.1 ± 38.7 mg/g; *p* = 0.023), and the MD-PA group (−8.5 ± 22.2 mg/g; *p* = 0.002) but not for the MD-HMF group (0.6 ± 9.5 mg/g; *p* = 0.343). The interaction between time and groups showed significance, as the largest reduction in UACR at 6 months was observed in those two groups (CD and MD-PA) with higher UACR levels at baseline. There were no differences in UACR changes between the three groups at 6 months after adjusting for baseline values.

**Figure 1:**
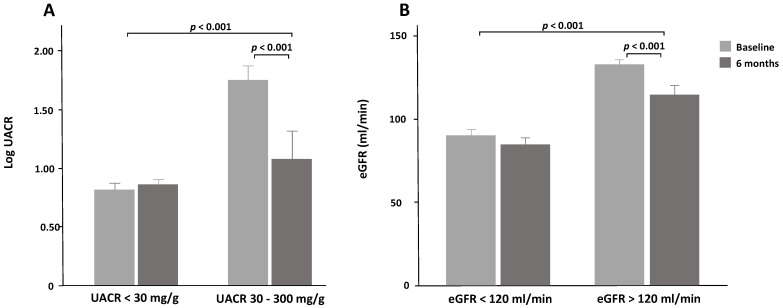
(**A**): Changes in urinary albumin-to-creatine ratio (UACR) between baseline and 6 months according to UACR baseline status. (**B**): Changes in estimated glomerular filtration rate (eGFR) between baseline and 6 months according to eGFR baseline status.

**Figure 2:**
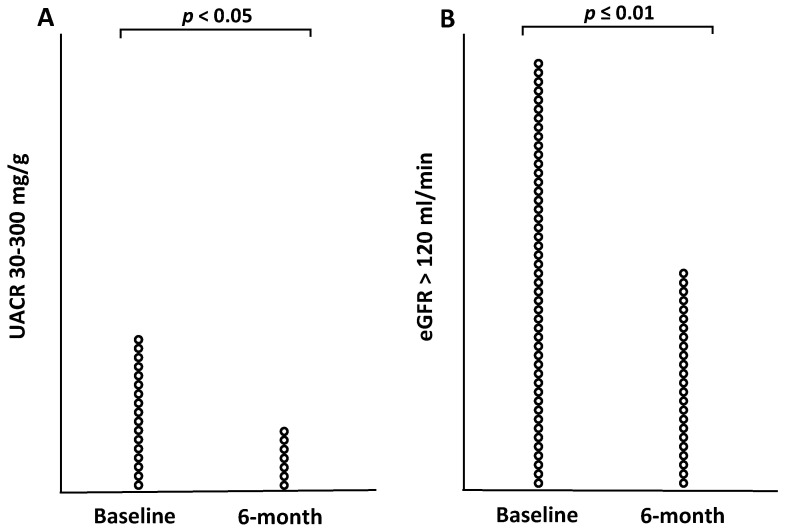
(**A**): Number or patients with increased albuminuria at baseline and 6 months. (**B**): Number of patients with hyperfiltration at baseline and 6 months.

When the subjects were stratified according to baseline stages of UACR (normal UACR < 30 mg/g, and moderately increased UACR = 30–300 mg/g), patients with moderately increased albuminuria experienced a significant reduction in UACR, whilst those with normal baseline UACR values experienced no change (Figure 1A and Table 2). The difference between patients with UACR < 30 mg/g and UACR 30–300 mg/g group controlled for baseline values was significant (*p* < 0.001), as well as the effect of interaction between stages of UACR and time on changes in UACR (data not shown). After performing a McNemar-Bower symmetry test, 14 (82%) of the 17 patients with baseline values of UACR between 30 and 300 mg/g reverted to a stage or normal albuminuria (*p* = 0.031) (Figure 2A). Out of the three intervention groups, the MD-PA and CD groups experienced the most significant reduction. In the MD-PA group, all of the 9 patients with increased albumin levels at baseline regressed to a normal state after 6 months (*p* < 0.001); in the CD group, 4 of 6 patients regressed to normal UACR. As for the MD-HMF group, only two patients (1.6% of the entire sample) presented increased albuminuria at baseline, and 1 regressed to a normal state of albuminuria.

Mean eGFR remained practically unchanged after 6 months intervention for the whole sample, as well as for the three intervention groups; between-group differences were also non-significant. Nevertheless, when dividing the sample according to eGFR values (normal filtration and hyperfiltration), those who showed hyperfiltration experienced a greater eGFR reduction compared to those who showed normal filtration (Figure 1B) and (Table 2). When controlling for baseline values, the difference between the two groups (normal and hyperfiltration) was significant (*p* < 0.001). The interaction between eGFR groups and time was also significant (*p* = 0.002). When performing a McNemar-Bower symmetry test, 18 patients (42.9%) reverted to normal filtration from a state of hyperfiltration (*p* = 0.004) (Figure 2B). Out of the three intervention groups, the MD-PA experienced the most reduction (12.1 ± 18.8 mL/min; *p* = 0.017), with 8 out of 17 patients with hyperfiltration at baseline regressing to normal filtration after 6 months.

At baseline, mean liver fat % was 13.4 ± 11.2, with 22 participants (17.2%) presenting severe steatosis, while liver stiffness was 5.4 ± 2.1 kPa, with 28 patients (21.9%) presenting fibrosis; no patients presented NASH. Mean liver enzymes were 37.5 ± 31.1 U/L for ALT, 26.2 ± 13.4 U/L for AST, and 19.2 ± 54.5 U/L for GGT. Following intervention, liver fat decreased for the whole sample and for the three groups. Differences in NAFLD changes were significant between the CD and MD-HMF groups (*p* = 0.030). Time per group interaction was not significant. Interestingly, although mean liver stiffness did not change for the whole group, nor for any of the intervention groups. Fifteen of the 28 patients that presented fibrosis at baseline reverted to a normal stage after intervention; however, this change was not significant. ALT, AST and GGT were significantly reduced in the whole group. ALT levels were reduced significantly in the three intervention groups, AST was reduced in the CD group only, and GGT was reduced in the CD and the MD-PA groups. No differences in changes in transaminase levels were observed between groups; time per group interaction was also not significant.

### 3.3. Effect of Intervention on Clinical and Biochemical Parameters

As shown in Table 3, after 6 months intervention, systolic BP (−5.6 ± 15.7 mmHg; *p* < 0.001) and diastolic BP (−3.9 ± 8.7 mmHg; *p* < 0.001), heart rate (−5.1 ± 8.8 bpm; *p* < 0.001), and mean arterial pressure (MAP) (−8.2 ± 20.3; *p* < 0.001) were significantly decreased for the whole sample and in each intervention group. Changes between groups were not significant for any of the considered parameters. Time per group interactions for systolic and diastolic BP, heart rate (HR), and MAP were not significant.

At baseline, fasting glucose for the whole sample was 116.2 ± 42.4 mg/dL, HbA1c was 6.1 ± 1.2%, and the average HOMA-IR was 5.9 ± 9.8. Fasting glucose was significantly decreased for the whole sample, as well as for the CD group, but did not change for the MD-HMF and the MD-PA groups. Fasting insulin was significantly reduced for the whole sample and for each of the interventions. HbA1c was significantly reduced for the whole sample and for the MD-HMF and MD-PA groups only. HOMA-IR was significantly decreased for the whole sample, as well as for each intervention. Likewise, TyG was significantly decreased for the whole sample, as well as for each group. Changes between groups and time per group interactions were not significant for any of the considered parameters.

Total cholesterol levels decreased significantly for the whole sample and for the MD-HMF group. HDL-cholesterol levels increased for the whole sample, as well as for the intervention groups. LDL-cholesterol levels, on the other hand, did not change. TG levels were reduced for the whole sample, as well as for the intervention groups. There were no differences in changes in blood lipid parameters between groups; time and group interactions were not significant.

Serum ferritin was reduced in the whole sample and in the CD and MD-HMF groups but not in the MD-PA group. No differences in changes in serum ferritin were observed between groups, and the interaction between time and group was also not significant. 

### 3.4. Effect of Intervention on Energy and Nutrient Intake

Energy intake significantly decreased for the whole group and within each intervention group (Table 4). However, between group analysis and time per group interaction did not show significance. In terms of macronutrient intake, the sample reduced carbohydrate and lipid intakes, while protein intake remained unchanged. The reduction in lipid intake was explained by a reduction in mono-unsaturated fatty acids (MUFA) (*p* = 0.010), saturated fatty acids (SFA) (*p* < 0.001), trans fatty acids (TFA) (*p* < 0.001), cholesterol (*p* < 0.001), and by a reduction in animal fat consumption (*p* < 0.001). Omega-3, on the other hand, was increased (*p* = 0.004). Differences in macronutrient intakes between the three groups were observed for carbohydrates only and were significantly reduced in the MD-HMF group compared to the CD group (*p* = 0.049). The combined effect for time and groups was not significant on any of the macronutrients considered.

As shown in Table 5, mineral and vitamin intakes were improved for the whole sample, and magnesium and potassium intakes increased, while that of sodium decreased. Vitamin B6 and folic acid intake were also increased. Differences in mineral and vitamin intakes between the three intervention groups were observed for sodium, which was significantly reduced in the MD-HMF group as compared to the others (*p* = 0.020): vitamin B6, which was significantly increased in the MD-HMF groups compared to the MD-PA group (*p* = 0.049); and vitamin D, which was significantly increased in the MD-PA group as compares to the MD-HMF group (*p* = 0.020). A combined effect for time and group was observed for potassium (*p* = 0.009), vitamin B2 (*p* = 0.047), vitamin B6 (*p* = 0.009), folic acid (*p* = 0.020), and vitamin C (*p* = 0.030).

Changes in macro- and micronutrient intakes were explained by an increased consumption of vegetables, fruits, legumes, nuts, and fish, and by a general increase in foods from vegetable sources, with a concomitant reduced consumption of cereals, meat and meat products, and sugary foods, such as sweets and pastries, as shown in Table 6. Differences between groups were observed in fruit consumption only, where the MD-HMF group significantly increased fruit consumption compared to the CD group (*p* = 0.030). Time per group interaction was significant for consumption of fruit (*p* = 0.005) and foods from vegetables sources (*p* = 0.010).

### 3.5. Correlation Analyses and Predictors of UACR and eGFR Reduction

Baseline UACR significantly correlated with BMI, systolic BP, TG, and sodium intake at 0.05 levels. UACR reduction significantly correlated with BMI (*p* = 0.030) and UACR (*p* < 0.001) as baseline values. When entered in the same multivariate regression model, baseline UACR remained the only significant predictor of changes in UACR (R^2^ = 0.492, *p* < 0.001). UACR reduction was also significantly correlated with an increase in omega-3 fatty acids consumption (*p* = 0.006). At multivariate analysis when delta of omega-3 fatty acids was adjusted for delta of energy intake, BMI, systolic BP, HbA1c, TG, physical activity, and intervention group, the association with changes in UACR was lost (data not shown).

Baseline eGFR significantly correlated with age, waist circumference, weight, and total fruits intake at 0.01 levels. eGFR reduction was correlated with baseline values of HbA1c (*p* = 0.032), eGFR (*p* < 0.001), animal protein (*p* = 0.019), dietary cholesterol (*p* = 0.008), and foods from animal sources (*p* = 0.040). At multivariate analysis, basal eGFR remained the only significant predictor or eGFR change (R^2^ = 0.227; *p* = 0.002) (data not shown). As shown in Table 7, eGFR reduction also correlated with reduction in mean liver fat % (*p* = 0.030), dietary cholesterol (*p* = 0.020), and with an increase in fruit consumption (*p* = 0.030). At multiple regression analysis, after adjusting for delta of energy intake, BMI, systolic BP, TyG-index, physical activity, and intervention group, changes in mean liver fat % (*p* = 0.007), TyG levels (*p* = 0.040), and physical activity (*p* = 0.030) were associated with changes in eGFR. 

## 4. Discussion

In the current randomized trial of patients with metabolic syndrome and NAFLD, 6-month weight loss intervention significantly ameliorated glomerular hyperfiltration and moderately increased albuminuria. Reduction of eGFR was associated with a reduction in liver fat % and TyG-index, as well as with an increase in physical activity. There were no differences between groups in neither UACR nor eGFR reduction. However, since the CD and the MD-PA groups showed higher levels of UACR at baseline, patients in these groups also experienced a higher decrease in UACR values. 

eGFR decreased by 7.35 ± 13.10% in patients presenting hyperfiltration at baseline. Similar results were found in a previous pilot intervention study on 70 hyperfiltration patients with diabetes and abdominal obesity, in which GFR was measured by iohexol plasma clearance [10]. In the intervention group with caloric restriction, GFR was reduced by 7.6 ± 11.7%, while, in the control group, it was reduced by 2.7 ± 11.1%, over a 6-month follow-up. Amelioration of hyperfiltration was associated with a reduction in blood pressure and body weight, and, most importantly, the short-term GFR reduction achieved by caloric restriction predicted a long-term GFR decline of 0.8 mL/min/1.73 m^2^ per month compared to the that of the control group, which predicted a faster long-term decline of 0.36 mL/min/1.73 m^2^ per month. Previous authors concluded that the amelioration of hyperfiltration was reno-protective as the predicted rate of GFR decline was similar to that observed in aging healthy adults [37]. Similarly, in a longitudinal study [8] including a cohort of 600 hypertensive T2DM patients with normal levels of albuminuria at baseline, a GFR reduction by 10% or more within a period of 6 months with angiotensin converting enzyme (ACE)-inhibitors was associated with a significantly slower GFR decline over 4 years. Amelioration of hyperfiltration has been associated significantly with improved blood pressure and glucose control. In the current study, only one-third (31%) of patients with hyperfiltration were diabetics; nevertheless, hyperfiltration is associated with a worse cardiometabolic profile compared to normal filtration subjects [38], and it has been proposed as an early marker of renal damage in metabolically unhealthy obesity [38,39]. Moreover, hyperfiltration has been recently associated with an increased risk of cardiovascular disease and all-cause mortality [40]. Thus, amelioration of hyperfiltration by weight loss might be beneficial for patients with obesity and MetS as they may achieve a persistent reno-protective effect over the long term and possibly reduce cardiovascular risk. Importantly, treatment strategies for hyperfiltration including blood glucose lowering medications, use of Angiotensin-Converted-Enzyme – Angiotensin-Receptor-Blockers (ACE-ARBs) therapy, controlled protein intakes and weight loss have been suggested for patients with diabetes [41]. In patients who are not yet in need of medications, but that might benefit from hyperfiltration reduction, weight loss through diet and exercise ameliorates several associated risk factors, even at subclinical levels, and might achieve a significant short-term eGFR reduction with long-term renal protection.

The reduction of eGFR was significantly associated with a reduction in liver fat % and TyG-index, and with an increase in physical activity. Although this finding is consistent with the fact that NAFLD and CKD share multiple cardiometabolic risk factors including insulin resistance and obesity [4], evidence of a direct association between hepatic steatosis and GFR is only available in patients with an already compromised renal function (GFR <60 mL/min/1.73 m^2^). NAFLD has been recognized as a risk factor for the development of CKD [3]; between 20 and 50% of patients with NAFLD have CKD, compared with 5–30% of patients without NAFLD [42]. Moreover, once CKD is present, its severity is associated with progression of NAFLD to fibrosis and NASH [3]. On the other hand, the association between hyperfiltration and NAFLD to this date has only been described in obese children. The study observed that in a cohort of 179 obese children aged 12–16 years with NAFLD confirmed by histological examination, 20% showed hyperfiltration (>136 mL/min/1.73 m^2^), and 15% had low GFR (<90 mL/min/1.73 m^2^). Compared with normal eGFR, hyperfiltration was associated with greater NAFLD activity score, independent of age, sex, ethnicity, obesity severity, T2DM, and medications [11]. In the current study, the finding that amelioration of glomerular hyperfiltration was driven by a reduction of intrahepatic fat could be explained by the fact that, in Non-Alcoholic Fatty Liver Disease (NAFLD) synthesis, very low density lipoproteins (VLDL) are increased, and, in turn, increased amounts of triglycerides are delivered to non-adipose peripheral tissues and organs, such as the kidney [43]. Intracellular lipid accumulation can generate a toxic environment in which lipid metabolites reduce mitochondrial function and increase oxidation and inflammation [44,45], which, at the kidney level, can possibly contribute to a state of glomerular hyperfiltration [44]. Of note, ectopic fat accumulation strongly correlates with insulin resistance, which was also reduced in our sample and significantly predicted reduction of hyperfiltration. This finding is consistent with previous observations that insulin resistance plays an important role in the development of glomerular hyperfiltration [38,46] and that increased glucose disposal rate (GDR), achieved either through caloric restriction [10], bariatric surgery [9], or medical therapy [8] is associated with amelioration of hyperfiltration. Interestingly, in the previously mentioned longitudinal study [8], a major finding was that 23% of patients which were hyperfiltrating at baseline did not improve after the first 6 months of intervention with Renin-Angiotensin-Aldosterone System (RAAS) blockers and showed a much rapid GFR decline (4.2 mL/min/1.73 m^2^ per year) during the 4 years of follow-up compared to those who ameliorated hyperfiltration (1.7 mL/min/1.73 m^2^ per year). Persistent hyperfiltration was strongly associated with severely lower GDR as assessed by the hyperinsulinemic euglycemic clamp, suggesting a powerful causative relationship. 

A significant increase in physical activity, indicating increased energy expenditure, was also associated with a decrease in eGFR. Physical activity and increased energy expenditure have long been associated with amelioration of obesity and associated cardiometabolic risk factors [47], of insulin resistance [48], and, more recently, of hyperfiltration in the general population [49]. Physical activity reduces central obesity, inflammation, and oxidative stress and, together with diet, is the first line of therapy for the prevention and amelioration of most obesity associated conditions [48].

Amelioration of glomerular hyperfiltration was independent of protein intake. High protein intakes have been observed to induce glomerular hyperfiltration [50]; nevertheless, in our study, mean protein consumption remained virtually unchanged during the intervention. Sodium intake, on the other hand, was significantly reduced after intervention; however, it was not associated with changes in eGFR. 

Studies on the possible amelioration of renal hyperfiltration through an intervention aimed at reducing liver fat accumulation in patients without particular liver complications are lacking. However, when lifestyle modifications were applied to patients with NASH, improvement of liver histology was associated with an increase in eGFR [51]. In an interesting review by Glass et al. [52], it is speculated that, since NAFLD is associated with risk factors that characterize other metabolically linked diseases, such as CKD, amongst others, amelioration of NAFLD could improve renal function. To this date, the only available treatment for amelioration of NAFLD is through lifestyle modifications. Weight gain has been associated with the development of NAFLD, as well as weight loss, through diet and exercise, with its remission [53].

Lifestyle intervention in this study also reduced mean UACR in patients with increased albuminuria. Such a finding is clinically relevant as increased albuminuria is an established risk factor for nephropathy and cardiovascular diseases (CVD) [54], and an emerging risk marker for all-cause mortality, cardiac abnormalities, cerebrovascular disease, and peripheral arterial disease in the general population [54,55], whereas reduction in UACR has been associated with reduced cardiovascular morbidity and mortality independently of blood pressure control [53]. Ibsen et al. [56] observed that, in hypertensive adults receiving either losartan or atenolol during a mean follow-up of 4.8 years, lowering of UACR resulted in a stepwise decrease in cardiovascular event rate (cardiovascular death, fatal and non-fatal myocardial infarction, and fatal and non-fatal stroke). Interestingly, the reduction in event rate associated with a reduction in UACR occurred for patients with increased albuminuria, as well as in those with normal albuminuria at baseline. In the current study, a significant reduction in UACR was only observed in those with increased UACR at baseline, whereas those with normal baseline levels did not experience an appreciable change. Nevertheless, 82% of patients with baseline values of UACR between 30 and 300 mg/g reverted to a stage or normal albuminuria. The reduction of albuminuria was not associated with changes in liver fat, as initially expected. Previous cross-sectional and cohort studies showed an association between NAFLD and increased albuminuria [57], which is speculated to be driven by insulin resistance [58]. The fact that current results differ from previous evidence, as discussed later, could be due to the small number of patients, as well as to the study design. 

Interestingly, reduction of UACR was associated with higher albuminuria levels at baseline; the same was observed for eGFR, in which reduction was associated with a baseline hyperfiltrating stage, meaning that, in these patients, at increased risk of accelerated renal function loss, CKD, and CVD, the benefit of weight loss and increased physical activity was more clinically significant. Changes in UACR and GFR were concomitant to a generally improved cardiometabolic state. By increasing energy expenditure, reducing caloric intake, and improving diet quality, adult patients with NAFLD and MetS achieved weight loss and reduced waist circumference, improved liver profile, blood pressure, glucose control, and blood lipid profile, and reduced insulin resistance. Taken together, these findings suggest that improving lifestyle habits can produce important cardiometabolic changes which could counteract the classical scenario of the evolution of renal function loss and obese nephropathy.

### Strengths and Limitations

The main strength of this study is that liver images were obtained by MRI, which is considered the most sensitive and accurate non-invasive method for quantifying liver fat [59,60,61]. Moreover, as shown by the reduction in weight and waist circumference and by the increase in energy expenditure, patients included in the study were highly compliant with the interventions. Contrary to what is usually argued, adherence to dietary and physical activity advice can be achieved, if done in a personalized manner and with motivating strategies. On the other hand, the major limitation was the secondary analysis design of the study. Patients were not included according to stages of UACR or GFR as inclusion criteria aimed specifically at studying patients with NAFLD and MetS, independently of possible renal involvement. Moreover, a bigger sample could give a more confident answer to the possible relationship between NAFLD and albuminuria/GFR in a population with MetS. 

## 5. Conclusions

In patients with NAFLD and MetS, lifestyle intervention significantly improved several major risk factors associated with accelerated renal function loss and CKD. Caloric restriction coupled with increased energy expenditure reduced hepatic fat accumulation and insulin resistance, which in turn significantly reduced glomerular hyperfiltration over a period of 6 months. Increased albuminuria was also significantly reduced; however, such change was not found to be associated with reduced liver fat. 

Patients with NAFLD are at increased risk of CKD, and hyperfiltration is the first stage of impaired function loss. Amelioration of NAFLD and hyperfiltration with lifestyle modification is a valid and inexpensive strategy for prevention of renal function loss and other associated conditions in patients with obesity and metabolic syndrome. Further evidence from long-term randomized clinical trials is needed to confirm the association between NAFLD and hyperfiltration and assess whether lifestyle intervention strategies can provide long-term renal, metabolic, and cardiovascular protection. 

## Figures and Tables

**Table 1:** Characteristics of the studied subjects.

	Whole Sample	CD	MD-HMF	MD-PA
*n*	128	42	43	43
Female	50 (39.1)	18 (42.9)	16 (37.2)	16 (37.2)
Of which having menopause	28 (21.9)	12 (28.6)	8 (18.6)	8 (18.6)
Age (y) (mean ± SD)	52.9 ± 7.4	54.1 ± 8.9	52.3 ± 7.1	52.2 ± 5.8
Currently smoking	24 (18.7)	6 (14.3)	9 (20.9)	9 (20.9)
Alcohol ≥ 7 drinks/week	25 (19.5)	7 (16.7)	9 (20.9)	9 (20.9)
Regular physical activity				
None	53 (41.4)	18 (42.9)	20 (46.5)	15 (34.9)
Light	47 (36.7)	12 (28.6)	12 (27.9)	23 (53.5)
Moderate	18 (14.1)	11 (26.2)	3 (7.0)	4 (9.3)
Heavy	9 (7.0)	1 (2.4)	8 (18.6)	-
T2DM	29 (22.7)	8 (19.0)	8 (18.6)	13 (30.2)
High BP	53 (41.4)	17 (40.5)	19 (44.2)	17 (39.5)

Abbreviations: BP: blood pressure; CD: Conventional Diet; MD-HMF: Mediterranean diet–high meal frequency; MD-PA: Mediterranean Diet–physical activity; SD: standard deviation; T2DM: type 2 diabetes mellitus. Data are expressed as count (%), unless otherwise specified.

**Table 2:** Changes in anthropometrical, lifestyle, renal, and hepatic variables within and between intervention groups at 6 months versus baseline.

		Whole Sample	CD	MD-HMF	MD-PA	t•g	*p* †
*n*		128	42	43	43		
Weight (kg)	Baseline	95.2 ± 13.5	92.4 ± 14.7	97.1 ± 14.2	96.0 ± 11.2	0.040	0.048 ^b^
	6 months	89.1 ± 13.3	86.7 ± 13.8	89.3 ± 14.3	91.1 ± 11.5		
	Δ	−6.1 ± 5.6 ***	−5.7 ± 5.5 ***	−7.8 ± 6.0 ***	−4.8 ± 5.0 ***		
WC (cm)	Baseline	111.8 ± 9.0	110.40 ± 9.19	112.7 ± 9.4	112.3 ± 8.4	0.130	0.150
	6 months	106.6 ± 13.7	107.08 ± 18.79	105.4 ± 12.0	108.0 ± 9.5		
	Δ	−5.2 ± 11.1 ***	−3.31 ± 17.04	−8.0 ± 5.8 ***	−4.3 ± 6.2 ***		
BMI (kg/m^2^)	Baseline	33.6 ± 3.6	33.4 ± 3.7	34.3 ± 3.9	33.2 ± 3.0	0.030	0.060
	6 months	31.5 ± 3.6	31.4 ± 3.7	31.6 ± 4.0	31.5 ± 3.3		
	Δ	−2.2 ± 2.0 ***	−2.0 ± 1.9 ***	−2.8 ± 2.1 ***	−1.7 ± 1.8 ***		
Fat mass (%)	Baseline	35.7 ± 7.0	35.9 ± 6.5	36.1 ± 7.1	34.9 ± 7.5	0.660	0.650
	6 months	34.3 ± 11.3	33.6 ± 7.2	35.2 ± 16.2	33.3 ± 8.0		
	Δ	−1.5 ± 9.2	−2.1 ± 2.9 ***	−0.4 ± 15.7	−1.9 ± 2.9 ***		
Physical activity (METs min/wk/100)	Baseline	30.1 ± 24.2	25.6 ± 20.1	33.4 ± 23.6	29.3 ± 27.6	0.850	0.780
	6 months	35.3 ± 24.2	34.6 ± 23.7	38.2 ± 23.6	33.0 ± 25.7		
	Δ	5.2 ± 26.2 *	7.2 ± 22.4	4.8 ± 27.7	3.7 ± 28.4		
MedDiet adherence	Baseline	7 ± 3	7 ± 3	7 ± 3	8 ± 2	<0.001	<0.001 ^a,b^
	6 months	12 ± 3	11 ± 3	13 ± 3	12 ± 3		
	Δ	5 ± 3 ***	4 ± 3 ***	7 ± 3 ***	4 ± 3 ***		
UACR ^# ‡^	Baseline	15.3 ± 31.8	21.1 ± 50.0	7.7 ± 8.6	17.3 ± 21.1	0.040	0.550
	6 months	9.0 ± 12.0	9.9 ± 13.9	8.4 ± 11.9	8.71 ± 10.37		
	Δ	−6.3 ± 26.5 ***	−11.1 ± 38.7 *	0.6 ± 9.5	−8.55 ± 22.2 **		
Normal UACR (<30 mg/g)	Baseline	7.2 ± 4.7	7.7 ± 5.1	6.0 ± 2.8	8.2 ± 5.8	0.890	0.930
	6 months	7.4 ± 8.5	6.9 ± 5.5	7.0 ± 8.4	8.4 ± 10.9		
	Δ	0.2 ± 8.8	−0.8 ± 6.50	1.0 ± 8.3	0.2 ± 11.4		
Moderately increased UACR (30–300 mg/g)	Baseline	68.1 ± 66.7	101.3 ± 106.4	44.1 ± 7.8	51.4 ± 23.2	0.240	0.260
	6 months	19.3 ± 22.9	27.9 ± 30.2	36.0 ± 38.2	9.9 ± 8.5		
	Δ	−48.8 ± 53.2 ***	−73.4 ± 81.1 *	−8.0 ± 30.3	−41.5 ± 22.5 ***		
eGFR (mL/min)	Baseline	103.0 ± 25.8	105.0 ± 24.4	101.2 ± 27.8	102.9 ± 25.5	0.910	0.980
	6 months	100.6 ± 25.3	101.8 ± 24.8	99.0 ± 28.3	100.9 ± 23.0		
	Δ	−2.4 ± 18.0	−3.2 ± 11.5	−2.1 ± 19.9	−2.0 ± 21.3		
Normofiltering (eGFR < 120 mL/min)	Baseline	89.2 ± 18.63	92.0 ± 18.1	88.9 ± 18.9	86.5 ± 19.2	0.440	0.620
	6 months	90.2 ± 20.3	90.6 ± 19.0	89.1 ± 22.0	91.1 ± 20.1		
	Δ	0.98 ± 17.73	−1.5 ± 8.3	0.2 ± 21.1	4.6 ± 20.6		
Hyperfiltering (eGFR > 120 mL/min)	Baseline	131.3 ± 11.3	130.9 ± 10.7	136.9 ± 16.5	127.9 ± 5.5	0.680	0.730
	6 months	121.8 ± 21.0	124.1 ± 19.6	128.0 ± 24.9	115.8 ± 19.0		
	Δ	−9.5 ± 16.7 ***	−6.8 ± 15.9	−8.8 ± 15.1	−12.1 ± 18.8 *		
Mean liver fat (%)	Baseline	13.4 ± 11.2	15.0 ± 10.1	12.2 ± 12.3	13.6 ± 11.4	0.680	0.030 ^a^
	6 months	8.0 ± 6.8	9.4 ± 7.7	5.6 ± 5.9	8.7 ± 6.1		
	Δ	−5.4 ± 9.4 ***	−4.9 ± 7.8 ***	−6.6 ± 10.6 ***	−4.9 ± 9.7 **		
Liver stiffness (kPa)	Baseline	5.4 ± 2.1	5.1 ± 1.9	5.4 ± 2.1	5.7 ± 2.4	0.290	0.360
	6 months	5.1 ± 1.6	5.301.7	4.8 ± 1.6	5.2 ± 1.5		
	Δ	−0.3 ± 2.3	0.2 ± 2.2	−0.6 ± 2.4	−0.5 ± 2.3		
ALT (U/L)	Baseline	37.5 ± 31.1	27.2 ± 10.5	40.55 ± 40.7	33.9 ± 28.1	0.360	0.560
	6 months	26.6 ± 17.4	26.7 ± 10.5	26.0 ± 13.1	27.0 ± 25.3		
	Δ	−2.8 ± 11.8 ***	−4.4 ± 8.6 ***	−3.72 ± 16.2 **	−0.1 ± 8.4 **		
AST (U/L)	Baseline	26.2 ± 13.4	37.7 ± 21.0	27.2 ± 17.7	23.6 ± 10.4	0.240	0.590
	6 months	23.4 ± 10.6	23.4 ± 6.3	23.5 ± 7.1	23.5 ± 15.9		
	Δ	−2.8 ± 11.8 *	−11.4 ± 17.2 **	−14.5 ± 34.5	−6.9 ± 17.9		
GGT (U/L) ^‡^	Baseline	49.5 ± 54.5	48.0 ± 31.1	62.3 ± 85.8	36.8 ± 15.8	0.690	0.660
	6 months	38.5 ± 51.3	34.6 ± 23.8	50.4 ± 82.7	30.3 ± 15.7		
	Δ	−10.9 ± 44.1 **	−14.7 ± 23.2 ***	−11.9 ± 71.7	−6.5 ± 10.8 ***		

Abbreviations: Δ: delta; ALT: alanine aminotransferase; AST: aspartate aminotransferase; BMI: body mass index; CD: Conventional Diet; eGFR: estimated glomerular filtration rate; GGT: gamma-glutamyl transferase; kPa: kilopascals; MD-HMF: Mediterranean diet–high meal frequency; MD-PA: Mediterranean Diet–physical activity; MedDiet: Mediterranean diet; METs: metabolic equivalents; UACR: urinary albumin-to-creatinine ratio; WC: waist circumference. Data are expressed as mean ± standard deviation (SD) and as count (% of the whole sample). # Log-transformed; ‡ Mean UACR and GGT were significantly different between the three groups at baseline. * *p* < 0.05; ** *p* ≤ 0.01; *** *p* ≤ 0.001 vs. baseline using the whole sample and within the same intervention group. t•g = time•group interaction. † Changes between the three intervention groups at 6 months after adjustment for baseline values by ANCOVA. a: Significant difference between CD and MD-HMF; b: Significant difference between MD-HMF and MD-PA.

**Table 3:** Changes in clinical and biochemical parameters within and between intervention groups at 6 months versus baseline.

		Whole Sample	CD	MD-HMF	MD-PA	t•g	*p* †
*n*		128	42	43	43		
SBP (mmHg)	Baseline	135.2 ± 14.6	137.5 ± 13.1	134.28 ± 13.9	133.4 ± 16.5	0.760	0.540
	6 months	129.6 ± 15.2	130.5 ± 16.2	127.7 ± 13.3	130.8 ± 16.0		
	Δ	−5.6 ± 15.7 ***	−7.0 ± 15.8 **	−6.6 ± 13.9 **	−3.2 ± 17.4 *		
DBP (mmHg)	Baseline	85.0 ± 8.8	84.1 ± 9.6	85.9 ± 7.8	84.3 ± 9.3	0.760	0.960
	6 months	81.0 ± 8.7	80.8 ± 8.8	81.2 ± 7.8	81.0 ± 9.7		
	Δ	−3.9 ± 8.7 ***	−3.3 ± 8.6 **	−4.7 ± 8.7 ***	−3.8 ± 9.0 **		
HR (bpm)	Baseline	70.3 ± 11.5	69.7 ± 11.1	68.9 ± 11.4	72.4 ± 11.7	0.450	0.790
	6 months	65.2 ± 10.4	64.6 ± 11.2	65.0 ± 10.1	66.2 ± 10.2		
	Δ	−5.1 ± 8.8 ***	−5.1 ± 8.6 ***	−3.9 ± 8.7 **	−6.4 ± 9.2 ***		
MAP	Baseline	191.5 ± 18.7	193.5 ± 17.4	191.6 ± 17.0	190.5 ± 21.6	0.620	0.670
	6 months	183.7 ± 19.6	184.4 ± 20.7	181.8 ± 16.9	184.8 ± 21.5		
	Δ	−8.2 ± 20.3 ***	−9.2 ± 20.1 **	−9.7 ± 18.6 **	−5.7 ± 22.5		
Glucose (mg/dL) ^#^	Baseline	116.2 ± 42.4	113.8 ± 33.1	109.7 ± 20.5	124.3 ± 61.7	0.500	0.880
	6 months	107.5 ± 38.7	107.7 ± 33.7	103.8 ± 35.5	111.0 ± 46.1		
	Δ	−8.7 ± 31.7 **	−6.7 ± 17.4 *	−5.9 ± 22.7	−13.3 ± 46.7		
Insulin (UI/mL)	Baseline	20.4 ± 10.8	22.3 ± 12.2	18.9 ± 9.8	20.1 ± 9.8	0.820	0.430
	6 months	15.4 ± 11.1	18.1 ± 13.3	14.1 ± 12.3	14.5 ± 6.3		
	Δ	−5.0 ± 9.6 ***	−4.2 ± 9.7 *	−4.9 ± 10.9 **	−5.6 ± 8.3 ***		
HbA1c (%)	Baseline	6.0 ± 1.2	6.0 ± 0.8	5.9 ± 0.9	6.3 ± 1.7	0.210	0.450
	6 months	5.8 ± 0.9	5.9 ± 0.7	5.7 ± 0.8	5.9 ± 1.2		
	Δ	−0.3 ± 0.8 ***	−0.1 ± 0.5	−0.2 ± 0.5 *	−0.4 ± 1.3 *		
HOMA-IR ^#^	Baseline	5.9 ± 3.8	6.4 ± 4.4	5.2 ± 3.3	6.2 ± 3.6	0.460	0.520
	6 months	4.5 ± 5.3	4.8 ± 3.7	4.4 ± 7.8	4.1 ± 3.1		
	Δ	−1.5 ± 4.5 ***	−1.6 ± 3.4 **	−0.8 ± 6.3 ***	−2.0 ± 3.3 ***		
Total-c (mg/dL)	Baseline	194.9 ± 39.3	202.0 ± 41.2	193.6 ± 36.5	189.5 ± 38.8	0.360	0.420
	6-months	188.2 ± 42.5	193.5 ± 45.8	182.1 ± 39.8	189.3 ± 42.2		
	Δ	−6.7 ± 37.5 *	−8.4 ± 30.0	−11.5 ± 34.0 *	−0.3 ± 46.2		
HDL-c (mg/dL)	Baseline	44.5 ± 10.6	44.9 ± 12.7	46.4 ± 9.1	42.2 ± 9.4	0.690	0.510
	6 months	46.9 ± 11.7	47.3 ± 13.6	49.4 ± 11.1	43.9 ± 9.8		
	Δ	2.4 ± 6.7 ***	2.5 ± 6.2 *	3.0 ± 8.6 *	1.7 ± 5.0 *		
LDL-c (mg/dL)	Baseline	115.4 ± 34.1	119.8 ± 37.6	116.2 ± 30.2	110.5 ± 33.7	0.130	0.180
	6 months	113.5 ± 37.1	116.4 ± 37.9	108.2 ± 35.9	116.7 ± 37.6		
	Δ	−1.9 ± 31.2	−3.8 ± 28.8	−7.6 ± 29.3	6.1 ± 34.5		
TG (mg/dL) ^#^	Baseline	181.1 ± 96.4	184.8 ± 87.3	160.6 ± 62.4	196.6 ± 126.1	0.970	0.170
	6 months	145.8 ± 93.0	150.9 ± 90.7	122.9 ± 68.7	163.9 ± 111.9		
	Δ	−35.3 ± 96.9 ***	−35.4 ± 71.2 ***	−37.7 ± 77.4 ***	−32.7 ± 131.6 *		
Ferritin (ng/mL) ^#^	Baseline	155.4 ± 153.7	161.9 ± 166.5	153.7 ± 151.0	150.7 ± 134.8	0.810	0.990
	6 months	124.0 ± 119.4	123.6 ± 112.1	124.8 ± 124.9	166.7 ± 227.9		
	Δ	−31.4 ± 96.8 **	−38.3 ± 103.9 *	−28.9 ± 95.4 *	21.2 ± 86.2		

Abbreviations: Δ: delta; CD: Conventional Diet; DBP: diastolic blood pressure; HbA1c: glycated hemoglobin; HDL-c: High density lipoprotein-cholesterol; HOMA-IR: Homeostatic Model Assessment for Insulin Resistance; HR: heart rate; LDL-c: Low density lipoprotein-cholesterol; MAP: mean arterial pressure; MD-HMF: Mediterranean diet–high meal frequency; MD-PA: Mediterranean Diet–physical activity; MedDiet: Mediterranean diet; SBP: systolic blood pressure; TG: triglycerides; TyG: triglycerides/glucose ratio; Total-c: total cholesterol. # Log-transformed. Data are expressed as mean ± standard deviation (SD). No significant differences were observed between the three groups at baseline. * *p* < 0.05; ** *p* ≤ 0.01; *** *p* ≤ 0.001 vs. baseline using the whole sample and within the same treatment group. t**•**g = time**•**group interaction. † Changes between the three intervention groups at 6 months after adjustment for baseline values by ANCOVA.

**Table 4:** Changes in energy and macronutrient intakes within and between intervention groups at 6 months versus baseline.

		Whole Sample	CD	MD-HMF	MD-PA	t•g	*p* †
*n*		128	42	43	43		
Energy (Kcal/d)	Baseline	2428.6 ± 810.1	2535.2 ± 612.7	2306.1 ± 1020.6	2455.8 ± 720.7	0.930	0.260
	6 months	2079.7 ± 619.6	2177.9 ± 680.3	1927.2 ± 515.7	2150.0 ± 644.5		
	Δ	−348.9 ± 848.6 ***	−357.3 ± 761.4 **	−378.9 ± 998.6 *	−305.7 ± 768.4 *		
CHO (g/d)	Baseline	252.4 ± 118.4	272.5 ± 77.2	237.4 ± 154.2	248.5 ± 107.2	0.790	0.049 ^a^
	6 months	212.1 ± 75.3	233.3 ± 73.3	187.7 ± 59.7	217.5 ± 86.7		
	Δ	−40.4 ± 117.0 ***	−39.2 ± 101.1 *	−49.7 ± 134.0 *	−31.0 ± 114.5		
Lipids (g/d)	Baseline	104.0 ± 38.5	105.6 ± 33.3	100.6 ± 44.1	106.4 ± 37.6	0.930	0.510
	6 months	87.9 ± 33.7	90.2 ± 35.5	82.3 ± 33.4	91.9 ± 32.3		
	Δ	−16.1 ± 44.3 ***	−15.4 ± 41.4 *	−18.3 ± 50.7 *	−14.5 ± 40.6 *		
Proteins (g/d)	Baseline	104.2 ± 29.2	109.5 ± 27.0	95.4 ± 26.8	108.9 ± 32.3	0.220	0.410
	6 months	100.5 ± 26.8	99.8 ± 30.8	95.9 ± 25.3	106.6 ± 23.1		
	Δ	−3.7 ± 26.4	−9.6 ± 25.4 *	0.6 ± 27.9	−2.3 ± 25.4		
Total fiber (g/d)	Baseline	25.0 ± 10.2	27.1 ± 8.5	22.6 ± 9.1	25.6 ± 12.6	0.100	0.220
	6 months	32.0 ± 11.7	30.9 ± 11.3	31.1 ± 10.0	34.1 ± 13.8		
	Δ	6.9 ± 11.1 ***	3.7 ± 10.5 *	8.5 ± 9.8 ***	8.5 ± 12.6 ***		
Alcohol (g/d)	Baseline	9.3 ± 14.3	8.2 ± 12.3	9.9 ± 12.6	9.8 ± 17.9	0.380	0.120
	6 months	5.4 ± 9.4	4.8 ± 7.7	7.4 ± 12.8	3.8 ± 5.6		
	Δ	−3.9 ± 11.1 ***	−3.3 ± 8.2 *	−2.5 ± 10.6	−6.0 ± 14.0 *		
Animal fat (g/d)	Baseline	53.2 ± 25.2	52.6 ± 13.6	51.7 ± 33.5	55.2 ± 23.1	0.430	0.090
	6 months	34.2 ± 18.6	40.5 ± 20.0	28.1 ± 19.0	35.8 ± 15.9		
	Δ	−19.0 ± 28.1 ***	−12.2 ± 22.7 *	−23.6 ± 36.7 **	−19.5 ± 21.3 ***		
Vegetable fat (g/d)	Baseline	62.5 ± 28.7	65.1 ± 30.1	60.8 ± 34.2	62.3 ± 22.0	0.860	0.580
	6 months	58.3 ± 29.9	62.7 ± 28.8	53.1 ± 33.3	60.2 ± 27.6		
	Δ	−4.2 ± 39.5	−2.4 ± 39.2	−7.7 ± 42.9	−2.1 ± 37.5		
MUFA (g/d)	Baseline	50.8 ± 19.7	52.3 ± 19.2	48.4 ± 21.7	51.9 ± 18.2	0.830	0.290
	6 months	44.9 ± 18.7	46.7 ± 19.2	40.9 ± 18.3	47.7 ± 18.3		
	Δ	−5.8 ± 24.1 *	−5.6 ± 24.5	−7.5 ± 26.2	−4.1 ± 21.8		
PUFA (g/d)	Baseline	17.4 ± 8.0	17.8 ± 6.5	15.9 ± 6.6	18.7 ± 10.5	0.190	0.360
	6 months	17.6 ± 10.4	15.9 ± 7.9	18.7 ± 13.6	18.2 ± 8.4		
	Δ	0.2 ± 11.8	−1.9 ± 8.2	2.8 ± 14.3	−0.5 ± 11.4		
SFA (g/d)	Baseline	28.5 ± 12.0	27.9 ± 9.1	28.1 ± 14.3	29.5 ± 12.0	0.470	0.270
	6 months	20.7 ± 10.0	22.2 ± 10.8	18.7 ± 9.6	21.3 ± 9.4		
	Δ	−7.8 ± 13.0 ***	−5.8 ± 10.8 **	−9.3 ± 15.6 ***	−8.2 ± 11.8 ***		
TFA (g/d)	Baseline	0.7 ± 0.4	0.6 ± 0.3	0.7 ± 0.4	0.8 ± 0.5	0.280	0.670
	6 months	0.4 ± 0.4	0.4 ± 0.3	0.3 ± 0.5	0.4 ± 0.4		
	Δ	−0.3 ± 0.5 ***	−0.2 ± 0.3 ***	−0.3 ± 0.6 ***	−0.4 ± 0.5 ***		
Cholesterol (g/d)	Baseline	431.7 ± 155.3	440.5 ± 168.3	407.9 ± 145.8	449.5 ± 152.7	0.890	0.640
	6 months	367.3 ± 147.4	366.2 ± 169.7	346.4 ± 144.2	392.5 ± 124.0		
	Δ	−64.3 ± 154.9 ***	−74.3 ± 144.6 **	−61.5 ± 173.4 *	−57.1 ± 146.7 *		
Omega-3 (g/d)	Baseline	0.9 ± 0.6	0.9 ± 0.6	0.8 ± 0.6	1.1 ± 0.6	0.320	0.080
	6 months	1.1 ± 0.7	1.0 ± 0.7	1.0 ± 0.6	1.3 ± 0.7		
	Δ	0.2 ± 0.6 **	0.1 ± 0.6	0.2 ± 0.6	0.3 ± 0.7 *		
Animal proteins (g/d)	Baseline	73.1 ± 21.5	76.9 ± 20.4	66.1 ± 18.5	77.4 ± 24.0	0.610	0.690
	6 months	67.9 ± 21.6	67.5 ± 24.0	63.5 ± 24.4	72.6 ± 16.0		
	Δ	−5.2 ± 21.6	−9.3 ± 24.3	−2.6 ± 22.5	−4.8 ± 18.7		
Vegetable proteins (g/d)	Baseline	34.9 ± 13.4	38.0 ± 10.1	33.3 ± 14.7	34.1 ± 14.4	0.360	0.340
	6 months	35.7 ± 13.0	35.3 ± 11.6	33.2 ± 13.4	38.6 ± 13.5		
	Δ	0.9 ± 16.5	−2.7 ± 14.3	−0.1 ± 17.5	4.5 ± 16.9		

Abbreviations: Δ: delta; CD: Conventional Diet; CHO: carbohydrates; MD-HMF: Mediterranean diet–high meal frequency; MD-PA: Mediterranean Diet–physical activity; MUFA: mono-unsaturated fatty acids; PUFA: poly-unsaturated fatty acids; SFA: saturated fatty acids; TFA: trans fatty acids. Data are expressed as mean ± standard deviation (SD). * *p* < 0.05; ** *p* ≤ 0.01; *** *p* ≤ 0.001 vs. baseline using the whole sample and within the same treatment group. t**•**g = time**•**group interaction. † Changes in between the three intervention groups at 6 months after adjustment for baseline values by ANCOVA. a: Significant difference between CD and MD-HMF.

**Table 5:** Changes in mineral and vitamin intakes within and between intervention groups at 6 months versus baseline.

		Whole Sample	CD	MD-HMF	MD-PA	t•g	*p* †
*n*		128	42	43	43		
Ca (mg/d)	Baseline	1045.8 ± 408.0	1089.6 ± 355.5	937.4 ± 351.2	1123.4 ± 496.0	0.230	0.760
	6 months	1048.2 ± 397.9	1036.7 ± 427.8	1032.0 ± 421.4	1078.8 ± 343.4		
	Δ	2.4 ± 426.1	−52.9 ± 429.5	94.6 ± 427.1	−44.5 ± 415.6		
Fe (mg/d)	Baseline	17.4 ± 6.1	18.8 ± 7.0	16.1 ± 5.1	17.5 ± 5.8	0.230	0.230
	6 months	17.9 ± 5.5	18.0 ± 5.8	16.8 ± 4.1	19.1 ± 6.3		
	Δ	0.5 ± 6.3	−0.9 ± 7.3	0.7 ± 5.5	1.7 ± 5.9		
Mg (mg/d)	Baseline	418.0 ± 130.6	440.9 ± 97.7	375.8 ± 133.1	442.1 ± 148.2	0.080	0.220
	6 months	472.8 ± 135.1	460.1 ± 135.3	458.4 ± 113.5	502.8 ± 155.1		
	Δ	54.8 ± 125.0 ***	19.2 ± 104.0	82.6 ± 127.7 ***	60.7 ± 136.0 *		
P (mg/d)	Baseline	1823.6 ± 541.3	1896.9 ± 436.5	1654.2 ± 532.3	19397 ± 613.7	0.210	0.320
	6 months	1915.0 ± 514.7	1878.6 ± 580.0	1829.0 ± 448.7	2051.9 ± 498.2		
	Δ	91.4 ± 481.9 *	−18.2 ± 416.5	174.7 ± 499.0 **	112.2 ± 516.9		
K (mg/d)	Baseline	4303.8 ± 1330.0	4623.3 ± 1055.0	3892.7 ± 1395.4	4435.9 ± 1426.4	0.009	0.060
	6 months	4664.0 ± 1273.8	4575.5 ± 1326.3	4570.8 ± 1088.9	4864.1 ± 1419.2		
	Δ	360.2 ± 1058.3 ***	−47.7 ± 709.9	678.1 ± 1257.4 **	428.2 ± 1002.2 *		
Se (μg/d)	Baseline	119.6 ± 41.5	125.2 ± 35.9	110.1 ± 41.7	124.5 ± 45.9	0.550	0.200
	6 months	114.4 ± 38.1	114.7 ± 39.9	105.1 ± 32.7	124.7 ± 40.2		
	Δ	−5.2 ± 41.1	−10.4 ± 36.6	−5.0 ± 39.3	0.2 ± 47.5		
Na (mg/d)	Baseline	2513.4 ± 954.6	2438.5 ± 753.7	2442.2 ± 1089.5	2674.0 ± 986.7	0.170	0.020 ^a,c^
	6 months	2053.1 ± 791.9	2173.7 ± 827.4	1765.5 ± 644.4	2254.4 ± 831.4		
	Δ	−460.3 ± 971.9 ***	−264.8 ± 866.8	−676.6 ± 900.8 ***	−419.6 ± 1122.1 *		
Zn (mg/d)	Baseline	14.2 ± 4.1	15.0 ± 3.7	13.2 ± 3.9	14.5 ± 4.5	0.570	0.400
	6 months	13.5 ± 3.8	13.7 ± 4.0	12.6 ± 3.0	14.2 ± 4.1		
	Δ	−0.7 ± 3.9	−1.2 ± 4.0	−0.6 ± 3.7	−0.3 ± 4.3		
Vit. A (μg/d)	Baseline	1201.5 ± 1081.9	1448.0 ± 1290.6	1144.7 ± 1169.4	1005.8 ± 616.5	0.260	0.820
	6 months	1110.9 ± 808.6	1182.3 ± 719.4	1104.7 ± 957.5	1042.5 ± 724.3		
	Δ	−90.6 ± 815.7	−265.7 ± 1149.7	−40.0 ± 579.9	36.7 ± 577.7		
Vit. B1 (mg/d)	Baseline	1.7 ± 0.7	1.9 ± 0.8	1.6 ± 0.6	1.7 ± 0.6	0.110	0.900
	6 months	1.8 ± 0.5	1.8 ± 0.5	1.7 ± 0.4	1.9 ± 0.6		
	Δ	0.1 ± 0.6	−0.1 ± 0.7	0.1 ± 0.6	0.2 ± 0.5 *		
Vit. B2 (mg/d)	Baseline	2.2 ± 0.9	2.4 ± 1.1	1.9 ± 0.7	2.2 ± 1.0	0.047	0.080
	6 months	2.3 ± 0.9	2.3 ± 1.0	2.1 ± 0.6	2.5 ± 1.1		
	Δ	0.1 ± 0.7	−0.1 ± 0.6	0.1 ± 0.7	0.3 ± 0.7 *		
Vit. B3 (mg/d)	Baseline	45.4 ± 14.0	48.6 ± 15.9	41.1 ± 11.6	46.9 ± 13.7	0.170	0.180
	6 months	44.4 ± 13.1	44.7 ± 14.1	41.0 ± 11.9	47.9 ± 12.8		
	Δ	−1.0 ± 11.8	−3.9 ± 11.8 *	−0.1 ± 12.7	1.0 ± 10.5		
Vit. B6 (mg/d)	Baseline	2.5 ± 1.0	2.8 ± 1.3	2.2 ± 0.7	2.6 ± 0.8	0.009	0.049 ^b^
	6 months	2.7 ± 0.8	2.7 ± 0.8	2.5 ± 0.7	2.9 ± 0.9		
	Δ	0.2 ± 0.8 *	−0.1 ± 0.9	0.3 ± 0.7 **	0.3 ± 0.6 **		
Vit. B12 (μg/d)	Baseline	11.0 ± 6.7	12.3 ± 7.8	9.8 ± 6.5	11.0 ± 5.4	0.180	0.340
	6 months	10.5 ± 5.2	10.4 ± 5.0	9.8 ± 5.9	11.3 ± 4.4		
	Δ	−0.5 ± 5.6	−1.9 ± 7.8	0.0 ± 3.9	0.3 ± 4.3		
Folic acid (μg/d)	Baseline	348.2 ± 154.0	387.0 ± 174.2	311.9 ± 129.5	348.7 ± 151.3	0.020	0.005 ^b^
	6 months	396.2 ± 134.0	380.9 ± 134.3	379.8 ± 100.8	431.1 ± 161.5		
	Δ	47.9 ± 145.8 ***	−6.1 ± 170.0	67.8 ± 132.9 **	82.4 ± 116.8 ***		
Vit. C (mg/d)	Baseline	194.7 ± 118.5	232.1 ± 142.6	164.8 ± 104.1	189.2 ± 96.3	0.030	0.200
	6 months	209.6 ± 84.9	209.7 ± 79.1	196.5 ± 67.5	224.3 ± 106.0		
	Δ	14.9 ± 104.1	−22.4 ± 127.3	31.7 ± 99.8	35.0 ± 67.5 **		
Vit. D (μg/d)	Baseline	6.6 ± 4.4	7.1 ± 5.2	5.6 ± 3.8	7.3 ± 4.1	0.180	0.020 ^c^
	6 months	7.4 ± 4.3	6.9 ± 4.2	6.1 ± 3.8	9.2 ± 4.6		
	Δ	0.7 ± 4.7	−0.2 ± 5.5	0.6 ± 4.1	1.9 ± 4.1 *		
Vit. E (mg/d)	Baseline	10.5 ± 5.1	10.5 ± 3.5	9.6 ± 4.0	11.7 ± 7.1	0.990	0.280
	6 months	10.4 ± 4.3	10.4 ± 4.6	9.5 ± 3.9	11.5 ± 4.4		
	Δ	−0.1 ± 5.9	−0.1 ± 4.2	−0.1 ± 5.9	−0.1 ± 7.4		

Abbreviations: Δ: delta; CD: Conventional Diet; MD-HMF: Mediterranean diet–high meal frequency; MD-PA: Mediterranean Diet–physical activity. Data are expressed as mean ± standard deviation (SD). * *p* < 0.05; ** *p* ≤ 0.01; *** *p* ≤ 0.001 vs. baseline using the whole sample and within the same treatment group. t**•**g = time**•**group interaction. † Changes in between the three intervention groups at 6 months after adjustment for baseline values by ANCOVA. a: Significant difference between CD and MD-HMF; b: Significant difference between CD and MD-PA; c: Significant difference between MD-HMF and MD-PA.

**Table 6:** Changes in food groups and food categories intakes within and between intervention groups at 6 months versus baseline.

		Whole Sample	CD	MD-HMF	MD-PA	t•g	*p* †
*n*		128	42	43	43		
Food groups							
Vegetables (g/d)	Baseline	307.3 ± 176.2	327.2 ± 196.79	285.2 ± 142.6	311.4 ± 189.7	0.210	0.180
	6 months	347.1 ± 168.3	331.8 ± 143.8	329.0 ± 141.9	384.0 ± 213.1		
	Δ	39.9 ± 162.6 **	4.6 ± 169.3	43.7 ± 174.8	72.7 ± 135.8 **		
Fruits (g/d)	Baseline	291.6 ± 201.5	329.5 ± 140.2	238.4 ± 201.9	312.5 ± 244.4	0.005	0.030 ^a^
	6 months	371.0 ± 228.6	343.9 ± 216.3	399.3 ± 238.9	367.3 ± 232.2		
	Δ	79.4 ± 209.2 ***	14.4 ± 204.2	161.0 ± 199.8 ***	54.8 ± 199.9		
Legumes (g/d)	Baseline	21.7 ± 11.9	22.2 ± 13.6	21.2 ± 11.4	21.9 ± 10.9	0.050	0.060
	6 months	34.0 ± 26.9	28.3 ± 19.6	41.4 ± 36.7	31.6 ± 17.5		
	Δ	12.3 ± 26.8 ***	6.1 ± 19.4 *	20.2 ± 36.2 ***	9.7 ± 18.2 **		
Cereals ^#^ (g/d)	Baseline	156.5 ± 99.9	169.3 ± 94.3	149.9 ± 99.0	150.6 ± 107.9	0.410	0.050
	6-months	127.8 ± 71.8	139.4 ± 67.9	105.3 ± 61.8	141.3 ± 81.4		
	Δ	−28.7 ± 114.4 **	−29.9 ± 111.2	−44.6 ± 108.8 *	−9.3 ± 124.1		
Milk and dairy (g/d)	Baseline	334.9 ± 202.2	365.1 ± 173.1	290.9 ± 172.1	353.2 ± 253.4	0.300	0.720
	6-months	360.0 ± 203.4	361.3 ± 220.1	355.2 ± 181.6	364.1 ± 214.4		
	Δ	25.1 ± 202.6	−3.8 ± 210.7	64.4 ± 171.5 *	10.9 ± 224.4		
Meat and meat products (g/d)	Baseline	182.4 ± 71.1	194.8 ± 79.2	172.7 ± 59.5	180.4 ± 74.4	0.740	0.960
	6-months	138.1 ± 69.3	142.9 ± 66.6	132.3 ± 81.7	139.7 ± 57.2		
	Δ	−44.3 ± 72.8 ***	−51.9 ± 79.8 ***	−40.4 ± 81.8 **	−40.6 ± 53.0 ***		
Olive oil (g/d)	Baseline	32.6 ± 19.8	34.9 ± 20.7	32.7 ± 22.4	30.1 ± 15.4	0.700	0.8700
	6-months	30.1 ± 18.1	31.1 ± 18.7	29.0 ± 17.3	30.4 ± 18.7		
	Δ	−2.1 ± 23.5	−3.8 ± 25.8	−3.7 ± 24.7	0.3± 19.5		
Fish (g/d)	Baseline	96.8 ± 61.3	101.9 ± 69.2	76.5 ± 43.2	114.5 ± 64.9	0.550	0.510
	6-months	125.1 ± 73.9	120.9 ± 85.2	110.5 ± 58.2	146.2 ± 74.2		
	Δ	28.3 ± 63.6 ***	18.9 ± 69.3	33.9 ± 54.0 ***	31.7 ± 67.9 **		
Nuts (g/d)	Baseline	11.6 ± 14.8	13.1 ± 14.8	7.4 ± 11.2	14.8 ± 17.5	0.090	0.200
	6-months	24.6 ± 28.7	19.1 ± 25.2	27.5 ± 34.9	27.0 ± 23.7		
	Δ	13.0 ± 29.0 ***	6.0 ± 26.1	20.3 ± 34.5 ***	12.1 ± 23.2 **		
Sweets and pastries (g/d)	Baseline	34.4 ± 59.7	28.9 ± 34.2	36.8 ± 80.9	37.7 ± 53.4	0.580	0.520
	6-months	14.2 ± 28.4	17.5 ± 31.9	10.3 ± 21.1	15.1 ± 31.9		
	Δ	−20.2 ± 65.0 ***	−11.3 ± 40.9 **	−26.4 ± 85.3	−22.6 ± 59.6 *		
Food categories							
Foods from animal sources (g/d)	Baseline	614.1 ± 244.7	661.9 ± 224.4	540.2 ± 192.8	648.1 ± 299.0	0.170	0.660
	6-months	623.3 ± 230.4	625.2 ± 270.1	598.1 ± 207.3	650.0 ± 213.0		
	Δ	9.1 ± 222.1	−36.8 ± 243.6	57.8 ± 184.4	1.9 ± 235.3		
Foods from vegetable sources (g/d)	Baseline	788.8 ± 332.1	861.3 ± 261.1	702.1 ± 309.4	811.1 ± 403.8	0.012	0.060
	6-months	904.6 ± 340.5	862.5 ± 307.5	902.8 ± 312.2	951.2 ± 403.0		
	Δ	115.8 ± 304.1 ***	1.20 ± 257.8	200.7 ± 304.6 ***	140.1 ± 318.9 *		

Abbreviations: Δ: delta; CD: Conventional Diet; MD-HMF: Mediterranean diet–high meal frequency; MD-PA: Mediterranean Diet–physical activity. Data are expressed as mean ± standard deviation. * *p* < 0.05; ** *p* ≤ 0.01; *** *p* ≤ 0.001 vs. baseline using the whole sample and within the same treatment group. t**•**g = time**•**group interaction. † Changes in between the three intervention groups at 6 months after adjustment for baseline values by ANCOVA. a: Significant difference between CD and MD-HMF. SD = standard deviation; # = excluding potatoes.

**Table 7:** Univariate and multivariate analyses of the association between change (delta) in eGFR (mL/min) and changes (delta) in possible covariates between baseline and 6 months for the whole sample (*n* = 128).

	Univariate Analysis		Multivariate Analysis		
Δ	*r*	*p*		SβC	*p*
			Model 1 ^a^		
Mean liver fat %	−0.210	0.030		−0.239	0.020
Dietary cholesterol (g/d)	−0.223	0.020		−0.190	0.050
Fruits (g/d)	0.211	0.030		0.242	0.014
			Model 2 ^b^		
Mean liver fat %				−0.298	0.007
Dietary cholesterol (g/d)				−0.138	0.350
Fruits (g/d)				0.198	0.070
Total Kcal (Kcal/d)				−0.058	0.700
BMI (kg/m^2^)				−0.049	0.680
SBP (mmHg)				−0.108	0.350
TyG-index				−0.234	0.040
Physical activity (METs min/wk)				0.239	0.030
Intervention group				−0.030	0.770

Abbreviations: Δ: delta; BMI: body mass index; METs: metabolic equivalents (min/week); r: correlation coefficient; SβC: standardized beta coefficient; SBP: systolic blood pressure; TG: triglycerides. ^a^ = R^2^ for the model = 0.146, *p* = 0.002; ^b^ = R^2^ for the model = 0.248, *p* = 0.006.

## Data Availability

There are restrictions on the availability of data for this trial, due to the signed consent agreements around data sharing, which only allow access to external re-searchers for studies following the project purposes. Requestors wishing to access the trial data used in this study can make a request to pep.tur@uib.es.
